# Annual Meeting of Constituents

**Published:** 1915-01-02

**Authors:** 


### METROPOLITAN HOSPITAL SUNDAY FUND.

### Annual Meeting of Constituents.

The annual meeting of constituents of this Fund was held at the Mansion House on Tuesday, December 22, the chair being occupied, in the unavoidable absence of the Lord Mayor, by the Vice-Chairman, Sir C. Ernest Tritton, Bart.

### The War and the Fund.

The Chairman, before asking the Archdeacon of Hampstead to move the first resolution, desired to remind constituents that since the last meeting the Fund had lost the services of Sir John Bell as Vice-Chairman, that gentleman having resigned in July, and he (the speaker) had been asked to assume the post *ad interim*; and as the Lord Mayor had found himself unable to attend, the presidency of this meeting fell to him automatically. The hospitals of London, about which those present were interested, had a still greater claim upon us to-day—great as that claim had always been—than at any previous time; and the reason was that within the wards of many of the London hospitals there were lying to-day numbers of wounded soldiers who had returned home from the battle front—men who had fought magnificently and heroically for their country in the hour of our need, men whose bravery was the talk of the world. It was a satisfaction to know that these men, who had added by their bravery fresh lustre to the glorious traditions of the British Army, were, on their return home, most carefully tended and looked after in the wards of our Metropolitan hospitals. The Report now presented was the first complete one since the lamented death of Sir Edmund Currie. It was then decided—and he believed it would be voted a wise decision—not to bring in an outsider as secretary, but appoint to the post one who had assiduously worked for the Fund, Mr. Arnold James, with Mr. J. A. R. Lander, who had also done much work in the same way, as assistant secretary. He had himself been in the closest touch with the Fund all through the year, during which there had not been a week, sometimes not a day, when he did not come to Mr. James, and he desired to bear public testimony to his efficiency.

We were now living in no ordinary times; we were on the edge of, perhaps, the saddest Christmas the world had ever seen; our land was deluged with sorrow. The House of Commons very wisely laid aside all prejudices and party passions; the voice of faction was hushed, and members of all views rallied as one man to the British Government to assist in carrying out this war to a successful issue. He felt that the same spirit of unanimity would characterise the proceedings at this meeting. In the sadness and solemnity of the time in which we were living, he hoped all would do the best they could to promote the interests of the hospitals, for whose sakes the constituents of this Fund were gathered together in this historic hall.

### The Income of the Fund.

The Ven. the Archdeacon of Hampstead moved: "That the Report of the Council for the year 1914 be, and is hereby, received and adopted." It was, he said, a difficult thing to stand in the shoes of the Bishop of London, who it was hoped would have been present to move the resolution, and who, in his inspiring way, would have encouraged his hearers to do more and more for the Fund, for the benefit of London's hospitals and dispensaries. It was a source of great congratulation that this year the total collected was £400 more than in the previous year, especially as, in the present exceptional circumstances, one could scarcely have wondered if it had happened to be somewhat less. He was sure the Bishop would have drawn a very encouraging message from that text. And his Lordship would have bidden them take the fact in a very humble and chastened spirit. Who could really be content with an income of something over £65,000 for the hospitals of which we were so proud? After all, it was surely but an inadequate amount. He hoped the public would be enheartened to give even more to such a splendid object, which so many people had so much at heart. There could be no question and no controversy about these splendid and marvellous institutions, and they deserved the fullest possible support, not as a matter of charity, but as one of right. And if that was so normally, how much more insistently did the demand come this year, with the difficult and expensive requirements which the hospitals were called upon to furnish in consequence of the war! The public should take care that there should be no anxiety with regard to the provision of adequate funds.

The Rev. Hardy Harwood, in seconding the resolution, said there were one or two items in the Report which should be briefly emphasised. He was sure the Council would not wish the reference to Sir John Bell's services to be passed over without a word spoken at the annual meeting. At the meetings of Council there had been every reason for those over whom Sir John presided to appreciate his uniform courtesy, as well as his devotion to the work of the hospitals. He felt sure he was expressing the feeling of all when he said the constituents were greatly indebted to him for the services he had rendered. It was a matter of satisfaction that the Fund had been able to fall back on Sir Ernest Tritton to take Sir John's place.

### A Change in the Report.

From the present Report there was absent a paragraph which had appeared for many years, when a certain number of churches were selected for mention, the criterion being those from which the largest sums had come in each denomination. It had been thought wiser to omit that, as it was sometimes a little invidious; the church which gave the largest amount was not of necessity the one which had the greatest devotion to the cause of the hospitals. As the proposer had said, it was very gratifying that so far the Fund had not been unfavourably affected by the various appeals connected with the war; though any such effect would naturally be felt next year instead of this. But he thought many would feel encouraged by the expectation that there would not be an adverse effect, because it was wonderful to see the generosity with which various special appeals had been met. Some societies had received even additional support since the commencement of the black days; people seemed to have increasingly awakened to the luxury of giving. It also taught the great lesson that what people wanted was to be convinced of the necessity of the object for which the appeal was made. There had certainly been a marvellous response to the nation's needs, both in lives and in money. Those who worked in provincial towns knew their hospitals well, and their support was everybody's business; it was only necessary to ask for money for them, and it would be forthcoming. But in

London there was not that immediate and intimate knowledge, and it was for the Council to consider whether the public were kept sufficiently informed as to what the Metropolitan hospitals were doing, though since they ministered to the sick and wounded from the war people generally had appreciated more how extensive their beneficent work was. At the end of a speech, asking for aid to hospitals, by one of our greatest surgeons, he told of a little child on whom he had operated, and whose eyes were bandaged. The child was anxiously groping about, when the nurse touched the child's hand, and at once its head fell contentedly back on the pillow. That little child's hand, said the surgeon, was the appeal of the hospitals on behalf of the sick poor of London to London's public. To those whose privilege it was to appeal from a pulpit for contributions to this Fund he suggested that, so far as possible, they should give first-hand information, not relying for material simply upon what was gathered from other people. That would constitute the most effective appeal which could be made. For the direct presentation of the merits of the appeal to the public the Fund was greatly indebted to the Press, and he hoped their good offices would be continued.

There was no response to an invitation to discuss the Report, and the resolution was put and carried unanimously.

ELECTION OF THE COUNCIL.

Sir WILLIAM CHURCH, Bt., K.C.B., M.D., moved: "That the Council for 1914 (including the Bishop of Rochester, the Bishop of Southwark, R.C., the Archdeacon of London, Rev. Master of the Temple, Rev. Prebendary Perry, M.A., Rev. J. Monro Gibson, D.D., Rev. R. J. Campbell, M.A., Rev. H. R. Gamble, M.A., Rev. Simpson Johnson, the Earl Cadogan, K.G., Lord Sandhurst, G.C.S.I., etc., Hon. Harry Lawson, M.P., Sir T. Vezey Strong, P.C., Sir Dyce Duckworth, Bt., M.D., etc., Herbert Brooks, Esq., George Drummond, Esq., F. H. Norman, Esq., and C. Evan Spicer, Esq., all of whom retire under Law VIII.) be and is hereby re-elected for 1915, with the addition of the Rev. Prebendary Eardley-Wilmot, M.A., the Rev. J. Gough McCormick, M.A., the Hon. W. H. Goschen, R. Ernest Alexander, Esq., Heath Harrison, Esq., and John Robarts, Esq., to fill vacancies." These names had been submitted by constituents, and were now recommended by the Council to this meeting of constituents to fill the vacancies.

The resolution was briefly seconded by the Rev. G. MARTIN, and carried unanimously.

HOSPITAL SUNDAY 1915.

The Rev. C. J. PROCTOR (Vicar of Islington) proposed: "That June 13 be fixed as Hospital Sunday in 1915, and that the cordial co-operation of all ministers of religion within the Metropolitan area be again invited in the usual way." He said he hoped the date named would be found generally acceptable to the churches, and that an even larger number than usual would be able to join in supporting such a worthy object. A further hope was that by that date the cloud of war might have been dispersed, and that, just as the collection for 1914 was made in a time of peace, so also would that of 1915. In any case, he felt sure that the spirit of generosity would fill people's hearts, if it were only as a memorial to the splendid work which the hospitals had done and were still doing in connection with the war. The dumb appeal mentioned by Mr. Hardy Harwood was clamant to-day, and it was very gratifying that the hospitals of London had risen so nobly to the great need; there had always been a universal need of hospitals among our people, and that need had now been greatly emphasised by the cruel exigencies of war. Everyone who had visited a relative in hospital must have come away moved by a spirit of gratitude that the requirement of the times was being met. We had cause to be proud of the noble institutions which had so admirably fulfilled their functions.

Sir DYCE DUCKWORTH, Bt., M.D., LL.D., seconded the resolution, expressing the hope that the sum subscribed on Hospital Sunday next year would be larger than ever before. The country had certainly learned to give in a manner which exceeded ordinary experience.

